# Regional variation in *Ascaris lumbricoides* and *Trichuris trichiura* infections by age cohort and sex: effects of market integration among the indigenous Shuar of Amazonian Ecuador

**DOI:** 10.1186/s40101-016-0118-2

**Published:** 2016-11-24

**Authors:** Theresa E. Gildner, Tara J. Cepon-Robins, Melissa A. Liebert, Samuel S. Urlacher, Felicia C. Madimenos, J. Josh Snodgrass, Lawrence S. Sugiyama

**Affiliations:** 1 Department of Anthropology, University of Oregon, Eugene, Oregon 97403 USA; 2 Department of Anthropology, University of Colorado, Colorado Springs, CO USA; 3 Department of Anthropology, Hunter College (CUNY), New York City, NY USA; 4 Queens College (CUNY), Flushing, NY USA

**Keywords:** Parasite infection, Indigenous health, Lifestyle change

## Abstract

**Background:**

Soil-transmitted helminth (STH) infection peaks during childhood and varies by sex. The impact of market integration (MI) (increasing production for and consumption from a market-based economy) on these infection patterns, however, is unclear. In this study, STH infection is examined by sex and age among indigenous Shuar inhabiting two regions of Amazonian Ecuador: (1) the modestly market-integrated Upano Valley (UV) and (2) the more traditional Cross-Cutucú (CC) region.

**Methods:**

Kato-Katz fecal smears were examined for parasite presence and infection intensity. Factorial ANOVAs and post hoc simple effects analyses were performed by sex to compare infection intensity between regions and age categories (infant/child, juvenile/adolescent, adult).

**Results:**

Significant age and regional differences in *Ascaris lumbricoides* and *Trichuris trichiura* infection were detected. Overall, infants/children and juveniles/adolescents displayed higher parasite loads than adults. CC females exhibited higher *A. lumbricoides* loads than UV females, while the opposite pattern was observed for *T. trichiura* infection in males.

**Conclusions:**

Regional infection patterns varied by sex and parasite species, perhaps due to MI-linked environmental and lifestyle changes. These results have public health implications for the identification of individuals at risk for infection and contribute to ongoing efforts to track changes and alleviate STH infection in indigenous populations undergoing MI.

## Background

The process of market integration (MI)—defined as increased production for and consumption from a market-based economy [1, 2]—affects the health of people throughout the developing world. Populations in transition from traditional subsistence production to more urban lifestyles generally experience rapid economic, social, and nutritional shifts including increased consumption of market foods, occupational changes, agricultural intensification, and increased mobility. Previous research demonstrates that these changes in daily activities and diet shape disease exposure and infection risk [2, 3]. Some of these changes (e.g., improved sanitation and healthcare practices) may lead to decreased pathogen exposure [4]. Conversely, other factors may increase parasite exposure, for example, MI appears to favor disease transmission through increasing population densities and facilitating the movement of unhealthy people and contaminated items between communities [4].

Research examining the health consequences of MI has focused predominantly on increased chronic disease rates [5, 6], largely ignoring how MI impacts infectious disease burden. Further, since chronic disease primarily affects adults, little is known about how MI affects health throughout the lifespan. Describing how and why infection patterns vary by age can clarify the threats posed at key developmental periods across MI levels, information useful for mitigating the substantial negative health impacts of these diseases. Infectious disease morbidity and mortality, particularly from parasitic infection, is disproportionally high among economically developing nations in tropical regions [7]. While some such diseases, like malaria, receive significant attention from western medicine [8], the so-called neglected tropical diseases (NTDs) do not, despite being responsible for approximately 534,000 deaths annually [9]. Furthermore, approximately 57 million life years are lost annually to premature disability and death caused by NTD infections, affecting over 1.4 billion people worldwide [9]. Soil-transmitted helminths (STHs) (parasitic worms), a common type of NTD, affect over one billion people in 149 countries [9, 10].

Research among the Amazonian forager-horticulturalist groups indicates that approximately 44–65% of participants are infected with at least one STH species [3, 11]. Chronic infection has been associated globally with several negative health consequences, including stunted growth, diarrhea, and nutritional deficiencies [12]. These infections can also result in major economic costs, including decreased work productivity and educational attainment [13–15]. Parasitic infection results in modified immune function (e.g., altered cytokine responses and T-cell profiles), often compromising the efficacy of vaccinations and impairing related health outcomes in STH endemic areas [16].

Research has consistently demonstrated that school-aged children harbor the highest STH loads [13, 15]. For instance, studies of children in Nigeria, Malaysia, rural China, and Madagascar report prevalence rates of approximately 45–89% for two of the most common STH infections (*Ascaris lumbricoides* [ascarids] and *Trichuris trichiura* [whipworm]), rates that appear to decrease with increasing age (e.g., Nigerian children exhibited *T. trichiura* prevalence rates 14% higher than older age groups) [17–20]. These high STH loads typically decrease over time as children exposed to endemic infection develop partial immunity [11, 14]. High infection rates in children have also been attributed to poor childhood sanitary habits, resulting in increased consumption of fecal-contaminated soil and child-to-child transmission [14].

Previous research further suggests that parasite infection risk may vary by sex in some cases [21–23], likely due to differences in developmental physiology and gender-related behavior. For example, females generally appear to mount more robust immune responses than males, including increased antibody secretion when the branch of immunity associated with parasitic infection is triggered [21, 22]. It has been proposed that this heightened immune response allows females to more effectively combat parasitic disease [23]. Males also appear to exhibit reduced immune function due to the immunosuppressive effects of the hormone testosterone [24] although this is not well tested in humans. Further, boys and girls may differ in their participation in activities associated with disease exposure. For instance, among the Nigerian schoolchildren, schistosomiasis exposure varies with gendered behaviors: boys are more likely to swim in infected rivers, while girls are more likely to be exposed through washing clothes or eating utensils [25].

Market integration can influence various social and lifestyle factors that, in turn, affect parasitic disease exposure. For example, economic changes often intensify reliance on cash crops, increasing disease risk through clear-cutting and the creation of standing water sources that serve as vector breeding sites [4]. These ecological changes increase the parasite infection risk by elevating the risk of human contact with parasitic disease vectors, such as mosquitoes [4]. Conversely, MI can decrease infection risk with improved community sanitation and healthcare access, as has been documented among the Tup´ı-Mondˆe of Brazil [4, 26]. Nevertheless, studies examining age- and sex-related STH infection patterns in relation to economic development within a single transitioning population are limited. These data are crucial for identifying the proximate factors most strongly associated with STH infection risk throughout the lifespan thus facilitating the design of effective prevention programs, especially where public health resources are limited.

The present study examines relationships among STH infection, age, and MI by sex among the Shuar, an indigenous population living in the lowland region of Amazonian Ecuador. The effects of MI on STH infection have been understudied among indigenous groups, and the Shuar represents a particularly useful study population given that they are currently experiencing rapid but regionally variable MI [1, 2]. Previous research indicates that STH infection is common among Shuar, with 65% of participants infected with at least one species [3]. In addition, infection is most common among subadults (<15 years of age), suggesting that age is an important disease determinant [3]. Furthermore, MI among the Shuar has been linked to a variety of health outcomes, including childhood nutritional status and adult cardiovascular health, suggesting that exposure to MI contributed to changes in population health [1, 2]. Analysis of age, sex, and regional variation in STH infection will contribute needed data for targeting interventions, both among the Shuar and other populations experiencing MI-related shifts in infectious disease. This study builds upon prior work with two objectives:

(i) Objective one: to compare STH infection across three developmental stages (infant/childhood, juvenile/adolescent, adulthood) by sex. Given high exposure rates, little acquired immunity, and relatively few physiological sex differences during early development, we predict that STH load will be highest among infants/children in both sexes.
(ii) Objective two: to compare STH intensity between two regions at different levels of MI across age groups, by sex. We predict that STH infection will be higher in more traditional communities for both sexes due to less stringent sanitation practices.

## Methods

### Study design

**Fig. 1:**
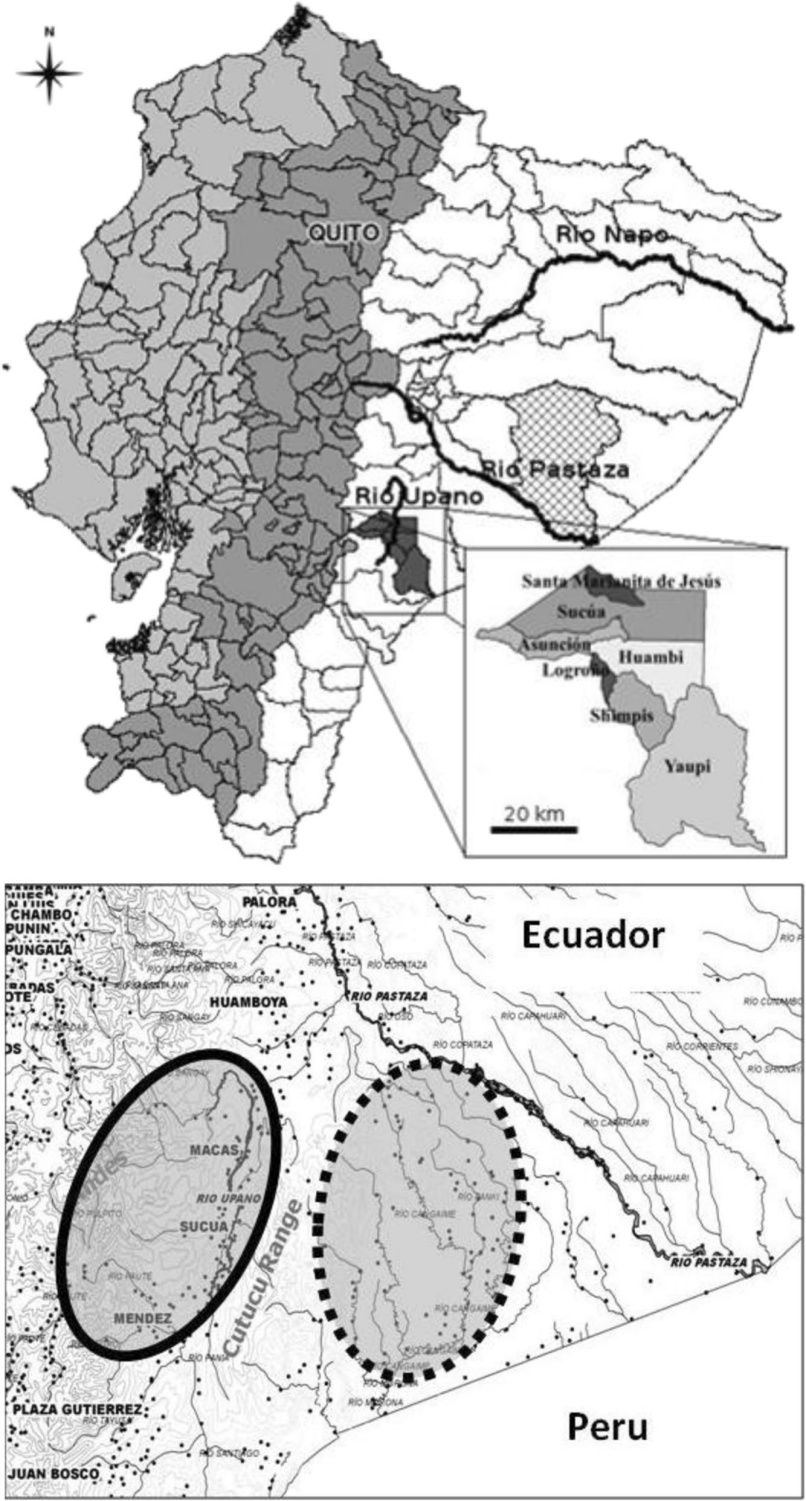
Map of Ecuador highlighting the Morona-Santiago region (*top*). Detailed depiction of the study area (*bottom*), including the Upano River Valley (*bold oval*) and Cross-Cutucú (*dashed oval*) regions. Image used with permission from Liebert et al. [21]

This study was conducted as part of the Shuar Health and Life History Project (SHLHP). The Shuar are an indigenous Amazonian population of ~40,000–110,000 individuals [27], primarily inhabiting the provinces of Morona-Santiago, Pastaza, and Zamora-Chinchipe, Ecuador (02°22′00″S 78°08′00″W; Fig. 1). Traditionally, Shuar lived in scattered households reliant on horticulture, fishing, hunting, and foraging [1, 27]. While MI is accelerating in the more remote region east of the Cordillera de Cutucú (Cross-Cutucú (CC)), market access for CC Shuar remains limited, and daily subsistence remains largely based on traditional practices [1]. In the more market-integrated Upano Valley (UV) region west of the Cutucú; traditional horticultural production is mixed with agro-pastoral production for market sale and various sources of wage labor.

#### Participants and sampling

The present cross-sectional study employed a regional comparative approach, with data collected over four field seasons (2011–2014). Significant differences were noted in infection rates across these years; however, these differences were generally between UV and CC communities, as expected. Random sampling was not feasible across this large population, dispersed over relatively inaccessible areas. Thus, through a collaborative effort with the *Federación Interprovincial de Centros Shuar* (FICSH), a sample of communities from the UV and CC was chosen, and all community members were invited to participate. The sample included 370 volunteers (197 females, 173 males) from three CC communities (*n* = 217; 114 females, 103 males) and two UV communities (*n* = 153; 83 females, 70 males) aged 6 months–100 years. Power analyses carried out prior to analyses indicated a sample of 251 individuals was required to adequately assess the main effects of age and region; the present sample size was therefore deemed sufficient to test the study hypotheses.

Ages were determined by birthdates on government-issued identification cards and cross-checked by informants and existing SHLHP genealogical data, as has been previously described elsewhere [1, 11]. To better examine differences in parasitic infection at developmental stages, individuals were divided into age groups, as outlined by Bogin [28]: infants/children (0–7 years old, *n* = 99), juveniles/adolescents (males 7–20 and females 7–17 years old, *n* = 125), and adults (males 21+ and females 18+ years old, *n* = 146).

### Field and laboratory procedures

#### Stool collection and analysis

Fresh stool samples were collected using the previously described methods [3]. A single Kato-Katz smear was prepared from each participant’s fecal sample (by either author TEG or TJC) within an hour of sample collection [29]; after 30–45 min, the smears were examined using ×10 and ×40 microscopy by trained observers (either TEG or TJC), and species-specific eggs per gram (EPG) (indicative of individual parasite load) of feces was recorded. *Ascaris lumbricoides* and *Trichuris trichiura* infections were detected: 25–53% of participants were infected with at least one of these helminths. The present study therefore focuses on these two species. While observers also looked for signs of hookworm infection (another type of STH), no hookworm ova were detected. Infection prevalence was calculated based on the proportion of participants with *A. lumbricoides* and/or *T. trichiura* ova in their stool. The mean natural log-transformed EPG values (lnEPG) were calculated for each age group, sex, and region to determine differences in infection intensity.

#### Market integration

Previous analysis of differences between individuals, households, and communities living in CC and UV indicate that average MI is greater in the UV than in the CC [1, 2]. Furthermore, significant differences in factors related to housing and socioeconomic status have been documented between these two regions [1, 2]. Specifically, a greater proportion of UV Shuar reported cinder block homes with concrete floors, bathrooms with running water, and domestic animal exposure in or around the house. UV Shuar also exhibited higher household income levels and increased consumption of processed food items than their CC counterparts [1, 2]. Conversely, more CC participants reported lacking designated latrine use, a greater reliance on river water, limited electricity, and living in houses constructed with wood walls and dirt floors [1, 2]. Region (UV vs CC) therefore provides a good proxy of general MI level.

### Statistical analyses

All analyses were conducted using SPSS version 20.0. Shapiro-Wilk tests were used to test for normality in species-specific EPG variables. As has been documented elsewhere, these values were not normally distributed and were therefore naturally log-transformed to achieve normal distributions [6]. Parametric tests of the transformed data were conducted to test the hypotheses.

(i) Descriptive statistics
  The number of participants infected with either *A. lumbricoides* or *T. trichiura* infection rates were calculated for each age category, by region and sex.
(ii) Examination of age and MI level differences
  Similar to other studies, all analyses were done separately by sex to account for potential differences in disease exposure and immune function [24]. Two 2 by 4 factorial ANOVAs were performed to test whether geographic region (indicative of MI level) and age category had significant main effects or displayed a significant interaction in relation to *A. lumbricoides* lnEPG values in males and females. Two additional 2 by 4 factorial ANOVAs were conducted to test the main effects and interaction of geographic region and age category in relation to *T. trichiura* lnEPG values, by sex. When significant interaction effects were found, post hoc simple effect tests with a Bonferroni adjustment (a technique used to examine the effect of one independent variable at individual levels of another independent variable [30]) were performed to test for differences in lnEGP values by age category within each region (CC and UV) and the effect of region within each age category. Given the small sample sizes apparent in some regional age/sex groups (e.g., UV adolescent males contained only four individuals), age categories were combined to maximize statistical power. This approach resulted in three final age categories: infants/children, juveniles/adolescents, and adults.

## Results

### Descriptive statistics

Table 1 presents infection counts by sex, age, and region. Overall, 47.0% of participants exhibited *A. lumbricoides*, and 32.4% exhibited *T. trichiura* infection (sexes, ages, and regions combined, *n* = 370). Males (ages combined) living in CC communities (*n* = 103) exhibited infection rates of 53.4% and 35.9% for *A. lumbricoides* and *T. trichiura*, respectively. Males living in UV communities (*n* = 70) exhibited infection rates of 42.9% and 38.6% for *A. lumbricoides* and *T. trichiura,* respectively. Females from CC communities (*n* = 114) displayed infection rates of 50.0% for *A. lumbricoides* and 24.6% for *T. trichiura,* while females residing in UV communities (*n* = 83) had infection rates of 38.6% for *A. lumbricoides* and 33.7% for *T. trichiura*.

**Table 1:** *Ascaris lumbricoides* and *Trichuris trichiura* infection counts and participant sample size within each age category, by region and sex

			*A. lumbricoides*		*T. trichiura*	
	Age category	Total (*N*)	Not infected (*n*)	Infected (*n*)	Not infected (*n*)	Infected (*n*)
CC males	Infant/child	**28**	14	14	17	11
	Juvenile/adolescent	**39**	19	20	24	15
	Adult	**36**	15	21	25	11
	Total	**103**	**48**	**55**	**66**	**37**
CC females	Infant/child	**29**	11	18	20	9
	Juvenile/adolescent	**35**	17	18	22	13
	Adult	**50**	29	21	44	6
	Total	**114**	**57**	**57**	**86**	**28**
UV males	Infant/child	**25**	14	11	13	12
	Juvenile/adolescent	**23**	12	11	9	14
	Adult	**22**	14	8	21	1
	Total	**70**	**40**	**30**	**43**	**27**
UV females	Infant/Child	**17**	11	6	10	7
	Juvenile/adolescent	**28**	18	10	16	12
	Adult	**38**	22	16	29	9
	Total	**83**	**51**	**32**	**55**	**28**

The bold values represent the total number present for each category

#### *A. lumbricoides* infection by region and age category

Geographic region had a significant main effect on *A. lumbricoides* lnEPG values in females only. CC females exhibited higher *A. lumbricoides* lnEPG values than UV females (mean difference = 1.63, *p* = 0.012). Conversely, age category did not have a significant main effect on *A. lumbricoides* lnEPG values in either sex (Table 2). A significant interaction between region and age category on *A. lumbricoides* lnEPG values was not documented for males and females (*p* = 0.248). However, post hoc simple effects analyses indicated that CC female infants/children exhibited significantly higher mean *A. lumbricoides* lnEPG values than their UV counterparts (*p* < 0.05) (Table 3).

**Table 2:** Post hoc pairwise comparisons of the difference in average natural log transformed *A. lumbricoides* and *T. trichiura* EPG values

			Raw mean values	Infants/children	Juveniles/adolescents
*A. lumbricoides*	Males	Infants/children	3.73	–	–
		Juveniles/adolescents	4.29	0.56	–
		Adults	3.39	−0.34	−0.90
	Females	Infants/children	4.36	–	–
		Juveniles/adolescents	3.63	−0.73	–
		Adults	3.12	−1.24	−0.51
*T. trichiura*	Males	Infants/children	2.29	–	–
		Juveniles/adolescents	2.65	0.36	–
		Adults	0.75	−1.53**	−1.89***
	Females	Infants/children	1.97	–	–
		Juveniles/adolescents	2.04	0.068	–
		Adults	0.82	−1.14*	−1.21**

Values represent the difference between each age category (separated by sex with regions combined), with a Bonferroni adjustment

The column listing age categories serves as the reference in each comparison

Comparisons are statistically significant at *p < 0.05; **p < 0.01; ***p < 0.001

**Table 3:** Pairwise comparisons of the difference in average natural log transformed *A. lumbricoides* and *T. trichiura* EPG values

		Infants/children	Juveniles/adolescents	Adults
*A. lumbricoides*	Males	4.28−3.17 = 1.11	4.71−3.88 = 0.82	4.37−2.42 = 1.95
	Females	5.80−2.92 = 2.88*	4.49−2.78 = 1.71	3.27−2.97 = 0.30
*T. trichiura*	Males	2.00−2.57 = −0.57	1.91−3.39 = −1.48*	1.29−0.22 = 1.07
	Females	1.65−2.29 = −0.65	2.00−2.07 = −0.07	0.52−1.12 = −0.60

Values represent the difference between CC and UV individuals (CC-UV), by age category (separated by sex) with a Bonferroni adjustment

Comparisons are statistically significant at *p < 0.05; **p < 0.01; ***p < 0.001

**Fig. 2:**
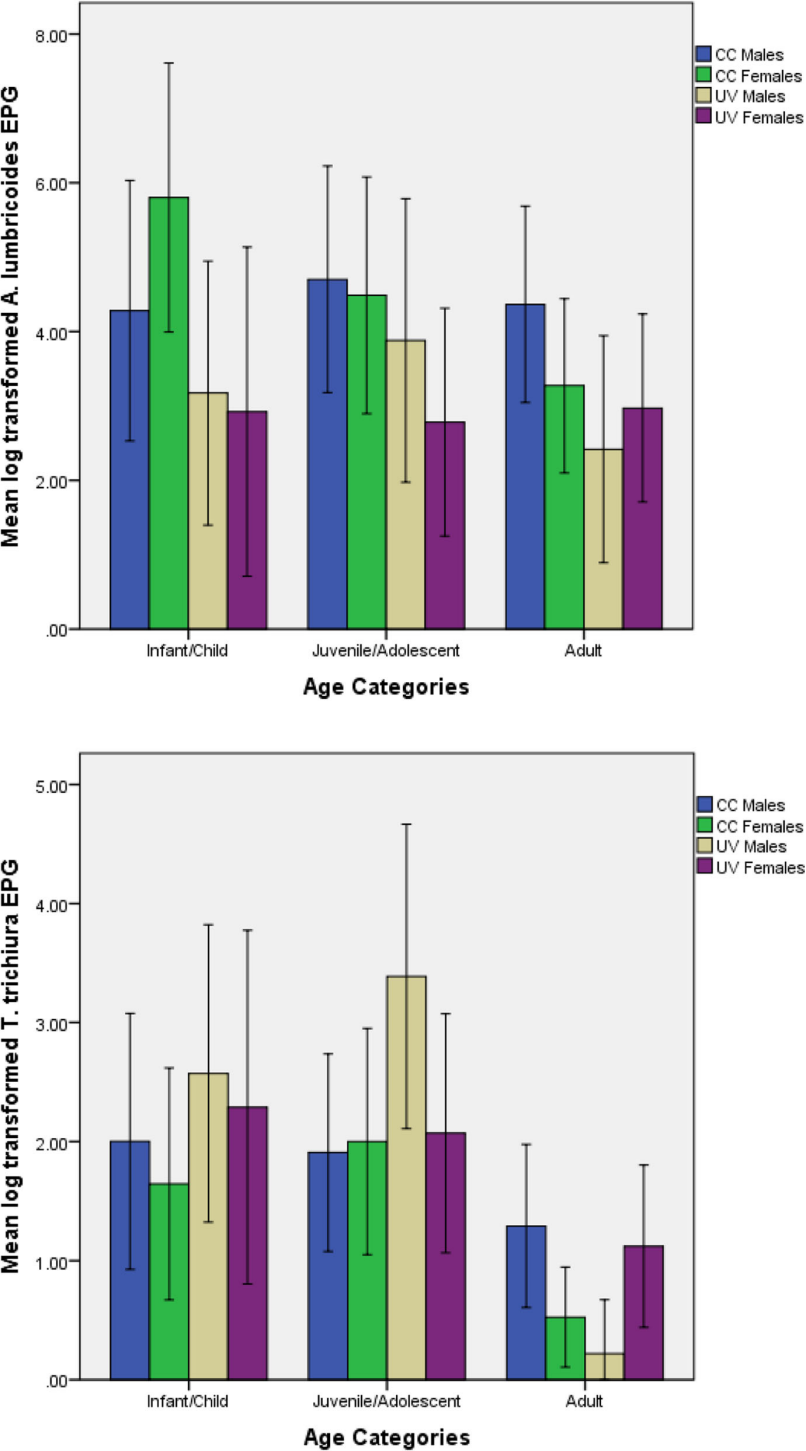
*A. lumbricoides* (*top*) and *T. trichiura* (*bottom*) natural log-transformed eggs per gram (EPG) values (with 95% CI error bars) by age category, region, and sex

Additional simple effects analyses were conducted separately by sex to examine how *A. lumbricoides* lnEPG level varied by age group within each region (Table 4). These tests indicated that CC female infants/children had significantly higher lnEPG values than CC adult women (*p* = 0.035). Figure 2 presents infection trends for *A. lumbricoides* by age category, sex, and region.

**Table 4:** Pairwise comparisons of the difference in average natural log transformed *A. lumbricoides* EPG values

		Raw mean values	Infants/children	Juveniles/adolescents
CC, males	Infants/children	4.28	–	–
	Juveniles/adolescents	4.70	0.42	–
	Adults	4.36	0.08	−0.34
CC, females	Infants/children	5.80	–	–
	Juveniles/adolescents	4.49	−1.31	–
	Adults	3.27	−2.53*	−1.22
UV, males	Infants/children	3.17	–	–
	Juveniles/adolescents	3.90	0.71	–
	Adults	2.42	−0.76	−1.46
UV, females	Infants/children	2.92	–	–
	Juveniles/adolescents	2.78	−0.14	–
	Adults	2.97	0.05	0.19

Values represent the difference for each age category by region type (separated by sex), with a Bonferroni adjustment

The column listing age categories serves as the reference in each comparison

Comparisons are statistically significant at: *p < 0.05, **p < 0.01, ***p < 0.001

#### *T. trichiura* infection by region and age category

Geographic region did not exhibit a significant main effect on *T. trichiura* lnEPG values in either sex. However, age category did have a significant main effect on *T. trichiura* lnEPG values in both males (*p* < 0.001) and females (*p* = 0.002). Post hoc multiple comparison analyses (using Bonferroni correction) assessed the main effect of age categories by sex (regions combined; Table 2). These comparisons indicated that male infants/children and juveniles/adolescents had higher lnEPG values than adult men. Similarly, female infants/children and juveniles/adolescents had higher lnEPG values than adult women.

A significant interaction between region and age category on *T. trichiura* lnEPG values was documented in males (*p* = 0.025) but not in females (*p* = 0.749). Given the significant interaction between region and age category in males, post hoc simple effects analyses were performed to examine regional differences in *T. trichiura* lnEPG values by age groups (Table 3). These analyses indicated that UV male juveniles/adolescents exhibited significantly higher mean *T. trichiura* lnEPG values than their CC counterparts.

Additional simple effects analyses were conducted separately by sex to examine how *T. trichiura* lnEPG level varied by age group within each region (Table 5). These tests indicated that UV male infants/children (*p* = 0.004) and juveniles/adolescents (*p* < 0.001) had significantly higher lnEPG values than UV adult men, and that CC juvenile/adolescent females (*p* = 0.013) displayed significantly higher lnEPG values than CC adult women. Figure 2 presents infection trends for *T. trichiura* by age category, sex, and region.

**Table 5:** Pairwise comparisons of the difference in average natural log transformed *T. trichiura* EPG values

		Raw mean values	Infants/children	Juveniles/adolescents
CC, males	Infants/children	2.00	–	–
	Juveniles/adolescents	1.91	−0.09	–
	Adults	1.29	−0.71	−0.62
CC, females	Infants/children	1.65	–	–
	Juveniles/adolescents	2.00	0.35	–
	Adults	0.52	−1.12	−1.48*
UV, males	Infants/children	2.57	–	–
	Juveniles/adolescents	3.39	0.81	–
	Adults	0.22	−2.35**	−3.17***
UV, females	Infants/children	2.29	–	–
	Juveniles/adolescents	2.07	−0.22	–
	Adults	1.12	−1.17	−0.95

Values represent the difference for each age category by region type (separated by sex), with a Bonferroni adjustment

Comparisons are statistically significant at: *p < 0.05, **p < 0.01, ***p < 0.001

The column listing age categories serves as the reference in each comparison

## Discussion

Significant age and regional differences in lnEPG values were observed for both *A. lumbricoides* and *T. trichiura* infections. As predicted, infants/children (and juveniles/adolescents in some cases) exhibited higher *T. trichiura* loads than adults in both regions, and *A. lumbricoides* infections were higher in CC female infants/children than their UV counterparts. However, in juvenile/adolescent males *T. trichiura* infection intensity was unexpectedly higher among the UV than the CC participants.

### Regional differences in STH infection

The high levels of *A. lumbricoides* infection among CC female infants/children is likely the result of increased contact with contaminated soil (e.g., children playing in unpaved community areas and homes) and the consumption of unfiltered water. These factors have been linked with increased exposure to *A. lumbricoides* in other populations [31, 32]. Additionally, differences in school antihelminthic treatment programs may account for regional infection differences in this age group. UV schools generally treat students several times a year, but regular in school treatment programs are less common in CC communities. School-aged CC children may therefore experience higher infection rates compared to UV school children.

Unexpectedly, UV male juvenile/adolescent participants exhibited higher *T. trichiura* lnEPG values than individuals living in CC communities. While contrary to our hypothesis, similar patterns have been documented elsewhere. Studies among Ecuadorian Cofán and Brazilian schoolchildren documented higher *T. trichiura* levels in more market-integrated areas than traditional communities, likely due to higher population density and poorly constructed houses [31, 33].

It is likely that a combination of environmental and lifestyle changes account for the high levels of *T. trichiura* infection evident among UV male juvenile/adolescent Shuar. For example, the increased movement of goods, livestock, and contaminated food items due to MI facilitates disease spread in highly populated areas [34]. UV Shuar have more regular access to market goods and livestock, and it is possible that young UV men may be exposed to *T. trichiura* infection via increased contact with domestic animals or travel to commercial areas while lacking the partial immunity evident in older men. While these potential explanations have not been tested, higher rates of animal contact around the house has been documented among UV Shuar [2], which may partially account for the high rates of infection observed in this age group.

The present results are generally consistent with earlier findings, which documented higher infection levels in CC than UV participants for general STH infection, coinfection, and *A. lumbricoides* infection but no relationship for *T. trichiura* [3]. The documented difference in *T. trichiura* infection is likely due to the inclusion of additional communities in the present study, specifically, one fairly market-integrated CC community and one UV community exhibiting a wide range of living conditions. These novel findings emphasize heterogeneity in the effects of MI on specific aspects of lifestyle (e.g., diet, latrine use, and house construction) across communities. Although regional comparisons are standard within epidemiological research [31, 33], this approach does not directly examine how local variation in MI patterns translate to the individual or household level and, in turn, influence parasite burden. Thus, while this study provides insight into Shuar infection risk across economic development, further data collection is needed to determine how particular lifestyle factors influence STH infection patterns.

### Age differences in STH infection

Previous work has documented high parasite loads among Shuar subadults (<15 years) [3] but did not determine how STH load varies during developmental periods. Here, we examined age cohorts in a larger sample of subadults to clarify these patterns. As expected, CC female infants/children had higher rates of *A. lumbricoides* than CC women. Similarly, infants/children of both sexes exhibited higher *T. trichiura* loads than adults in both regions. This supports previous findings [11, 14] and suggests that infection peaks during infancy/childhood in populations exposed to endemic parasitic infection and decreases with the development of partial immunity and improved hygiene practices [11, 14]. High infection levels during childhood may have especially severe consequences, including stunted growth and impaired cognitive development, which negatively impact lifelong health [12–15]. Shuar physical growth during childhood has been shown to be slower than that of industrialized populations [35, 36], with Shuar childhood body size demonstrating a close association with MI variables [2]. In addition, high rates of stunting (~40%) have been documented among Shuar children [37], suggesting that MI-induced patterns in diet and infection negatively affect Shuar growth and health. It is therefore critical that STH treatment programs focus on this vulnerable age group.

### Regional age-related STH infection patterns by sex

No significant age or regional differences in *A. lumbricoides* infection were documented in male participants, but were apparent in females; specifically, female infants/children had higher parasite loads than adults in the CC region. It is possible that CC female infants/children experience greater exposure to *A. lumbricoides* infection through their activity patterns. Shuar female children are more often observed assisting in childcare and household duties, potentially increasing disease risk through contact with infected children or contaminated food items. These possibilities remain untested, but previous work indicates that participation in gendered activities is associated with disease exposure and that males are preferentially treated for infection over females [25, 38, 39].

The same differences in behavior and medical treatment could also account for the high *T. trichiura* infection rates observed in CC female juveniles/adolescents (in comparison to adults) but not males. However, the opposite *T. trichiura* infection pattern was documented in males. UV male infants/children and juvenile/adolescent, but not CC males, exhibited higher parasite loads than adult men. This infection could be the result of MI-linked differences in behavior and physiology. For example, it is possible that MI-induced lifestyle changes alter sex hormone levels, with effects on immunity. Men in industrial populations typically exhibit significantly higher levels of the sex hormone testosterone than men in traditional subsistence communities [40]. This has been attributed to cultural differences in diet, physical activity, and a variety of other factors affecting testosterone [40]. In turn, high testosterone appears to have several immunosuppressive effects, including reduced immune cell activity [24]. MI may therefore lead to elevate testosterone levels, reduce immune function, and increase STH infection risk [40]. Current research among the Shuar is testing these hypothesized associations.

It remains unclear which of the hypothesized causes are responsible for the unpredicted higher rates of *T. trichiura* infection among more market-integrated males. Research examining disease patterns among transitioning, vulnerable populations is especially important given the long-term economic and health consequences associated with STH infection (e.g., limited worker productivity and educational attainment due to prolonged infection) [12–15]. Further work is needed to clarify which factors are most strongly linked with STH exposure and better control disease risk, thereby promoting optimal physical health and economic growth among these populations.

### Limitations

The present study has several limitations. First, small sample size in some regional age/sex groups limits the generalizability of these findings. Age groups were collapsed to reach the sample sizes required for analyses, precluding a more nuanced examination of infection differences between each of the five age categories. In addition, previous work shows significant variation in MI within as well as between regions (e.g., differences in house construction, water use, and healthcare), limiting the usefulness of regional comparisons. Future research with larger sample sizes will therefore investigate how particular features of MI affect STH infection risk within participant communities. Despite these limitations, the living conditions and high parasite load characteristic of the Shuar provide a unique opportunity to study STH infection patterns among a population experiencing MI.

## Conclusions

This preliminary study examined the association between MI and STH infection patterns across the lifespan in an unstudied setting, contributing unique data toward an improved epidemiological understanding of factors influencing parasitic disease risk. Results suggest that age and MI influence STH infection among the Shuar. Both *A. lumbricoides* and *T. trichiura* load varied between age groups by sex, perhaps due to MI-linked environmental and lifestyle changes. These results have implications for the identification of individuals at risk for parasitic infection. An improved understanding of parasitic disease patterns among the Shuar will not only facilitate the treatment of infected individuals, but will enable healthcare officials to better predict future infection likelihood based on patient sex, age, and region of residence. Finally, these findings may help track changes in infection risk and alleviate the high STH burden characteristic of indigenous populations undergoing MI.

## Abbreviations

CC: Cross-Cutucú region
EPG: Eggs per gram
lnEPGs: Mean natural log-transformed eggs per gram values
MI: Market integration
NTD: Neglected tropical diseases
SHLHP: Shuar Health and Life History Project
STH: Soil-transmitted helminth
UV: Upano Valley region
